# Integrating Internet of Things into cardiac rehabilitation for heart failure: a review of emerging technologies

**DOI:** 10.3389/fmed.2025.1737523

**Published:** 2026-01-23

**Authors:** Adriana Klein, Rafael Fernandes Pinheiro, Rui Fonseca-Pinto

**Affiliations:** 1 Center for Innovation Care and Health Technology (ciTechCare), Polytechnic of Leiria, Leiria, Portugal; 2 Instituto de Telecomunicações (IT), Leiria, Portugal

**Keywords:** cardiac rehabilitation, digital health, heart failure, Internet of Things, medical industry

## Abstract

Heart failure (HF) is a prevalent and debilitating condition that significantly affects patients' quality of life and places a substantial burden on healthcare systems. In recent years, digital technologies have been increasingly explored in cardiac rehabilitation (CR), particularly through their integration within Internet of Things (IoT) ecosystems to support remote monitoring and personalized care. This review aimed to provide a focused overview of emerging digital technologies applicable to the rehabilitation of patients with HF, with emphasis on solutions compatible with IoT-based systems. A targeted literature search was conducted in PubMed, Scopus, Cochrane and Web of Science, including studies published between 2019 and 2024. Studies addressing digital technologies in HF rehabilitation. Following a structured selection process, 59 articles were included in the narrative synthesis. The findings indicate a growing body of literature investigating wearable physiological monitoring devices, telehealth-based CR programs, digital platforms, and smart sensors, many of which have been explored for integration within IoT infrastructures. These technologies have been associated with improved remote follow-up, patient engagement, and real-time physiological data collection outside traditional clinical settings. Emerging applications of artificial intelligence within IoT-enabled systems have also been examined to support clinical workflows and adaptive rehabilitation strategies. Despite increasing interest, challenges remain, including heterogeneity in study designs, usability concerns, data privacy and security issues, economic barriers, and limited large-scale clinical validation. Overall, this review suggests that IoT-enabled technologies represent a promising area of research in CR, warranting further investigation to support their sustainable integration into routine HF care.

## 1 Introduction

Heart failure (HF) is a chronic condition that imposes a significant global health burden, affecting millions of individuals worldwide and contributing to high rates of hospitalization, morbidity, and mortality (1). Cardiac rehabilitation (CR) is a comprehensive, multidisciplinary intervention shown to improve functional capacity, alleviate symptoms, and enhance quality of life (QoL) in patients with HF (2). However, despite its proven benefits, CR remains widely underutilized due to challenges such as limited accessibility, low patient adherence, and healthcare resource constraints (2, 3). In recent years—particularly in the aftermath of the COVID-19 pandemic (4, 5)—the healthcare sector has experienced a surge in the development and adoption of emerging digital technologies. Among these, Internet of Things (IoT) systems have gained prominence for their capacity to connect wearable devices, remote monitoring tools, and mobile health (mHealth) applications, creating integrated networks capable of real-time data exchange. Combined with artificial intelligence (AI), virtual reality (VR), and robotic systems, these technologies have the potential to transform traditional models of CR into more accessible, personalized, and continuous care solutions (2, 3, 5). Given the increasing relevance of IoT and other digital innovations in cardiac care, it is essential to understand how such technologies are being implemented in CR for patients with HF. This review aims to identify and characterize emerging technologies—particularly those integrated with Internet of Things (IoT) systems—that are being developed or already applied to support cardiac rehabilitation in patients with heart failure. A scoping review methodology was adopted to explore the breadth and specific characteristics of the available literature, identify knowledge gaps, and guide future research in this evolving field.

## 2 Cardiac rehabilitation technologies and the role of the Internet of Things (IoT)

Over the past decade, a variety of digital technologies have been integrated into the rehabilitation of patients with heart failure (HF), including wearable devices, telemonitoring systems, artificial intelligence (AI), machine learning algorithms, mobile health (mHealth) applications, virtual reality (VR) platforms, and robotic systems (2–4). Among these, the Internet of Things (IoT) stands out as a transformative framework that connects these technologies through communication protocols such as Bluetooth and Wi-Fi, enabling seamless data transmission and remote patient monitoring. The widespread adoption of smartphones and wireless connectivity has opened new opportunities for the remote and real-time management of HF, allowing healthcare providers to monitor clinical parameters and adjust treatments without the need for face-to-face interaction. These IoT-based strategies have emerged as promising tools to reduce hospital readmissions, support self-management, and improve long-term clinical outcomes (2). Although several technologies have shown positive effects on clinical endpoints, further large-scale randomized controlled trials are necessary to validate their efficacy and assess long-term sustainability within healthcare systems (5). Clinicians must also remain cautious of the potential digital divide, as these innovations may inadvertently exacerbate health disparities among vulnerable or digitally excluded populations. A major innovation within this context is the implementation of remote monitoring systems, including videoconferencing, mobile apps, and cloud-based digital health platforms, which significantly enhance access to cardiac rehabilitation (CR) for patients in rural or underserved areas or those with physical limitations (2, 5). Wearable IoT-enabled devices such as smartwatches and heart rate monitors allow for continuous tracking of physiological parameters—including heart rate, blood pressure, and physical activity—enabling personalized, data-driven care and timely clinical interventions. Advances in AI and machine learning, when integrated with IoT systems, further enhance CR programs by enabling predictive analytics that assess risk, track treatment adherence, and forecast health outcomes. These intelligent tools support clinical decision-making and facilitate the creation of highly personalized rehabilitation pathways. Additionally, VR and AR technologies, often integrated into IoT ecosystems, have been explored for delivering supervised and interactive exercise programs, which enhance patient engagement and adherence. Collectively, these innovations are reshaping the landscape of cardiac rehabilitation by making it increasingly personalized, accessible, and responsive to individual patient needs. However, despite their therapeutic potential, large-scale implementation of these technologies remains limited—particularly among elderly people, who comprise the majority of individuals affected by heart failure. To fully realize the benefits of these technological advancements, it is essential to address barriers related to digital literacy, device cost, and usability, thereby ensuring equitable and sustainable adoption (5).

## 3 Objectives of the review

Given this scenario, the present review aims to investigate the integration of IoT and other emerging technologies in the rehabilitation of patients with heart failure and to identify innovation opportunities for the medical industry in this rapidly evolving field. The guiding research questions are:

**What emerging technologies are being applied in the rehabilitation of patients with heart failure?**

**How can IoT and emerging technologies transform traditional cardiac rehabilitation models and shape the future of care for heart failure patients?**

## 4 Methods

**Figure 1:**
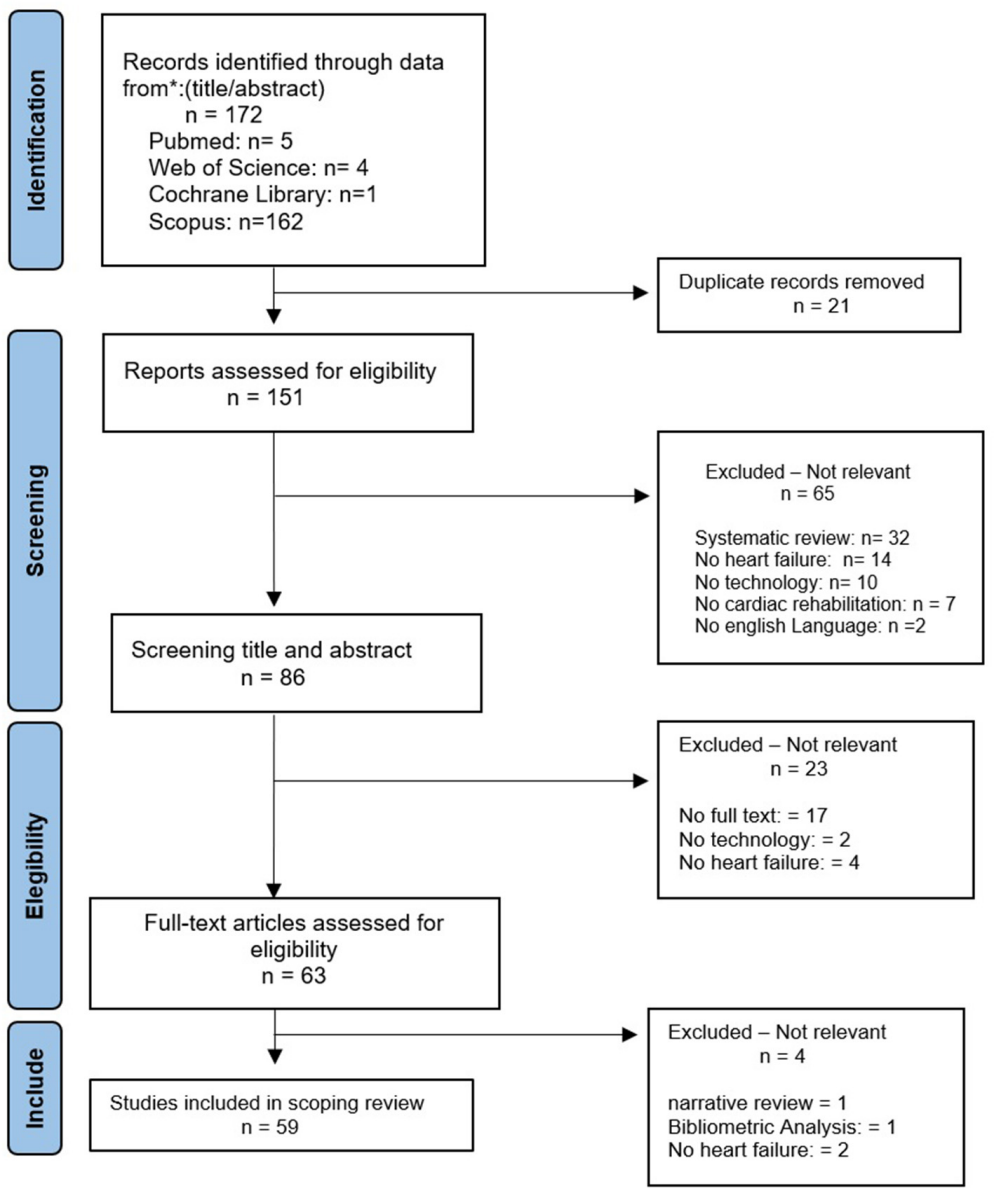
PRISMA flow diagram of the study selection process, detailing the number of records identified, screened, assessed for eligibility, and included in the final review.

The method applied in this review was based on a targeted and structured literature search designed to identify recent and relevant studies addressing the integration of digital technologies into CR for HF. A research strategy was defined a priori to guide the selection of studies, including the identification of relevant technologies, types of interventions, and reported outcomes. a PRISMA flow diagram was included to enhance transparency and facilitate visualization of the study identification and selection process (6). The protocol defined the inclusion and exclusion criteria, types of studies, identification of technologies, types of interventions, and outcome measures. The data used in this study were retrieved from PubMed, Cochrane Library, Scopus, and Web of Science. The search strategy combined terms related to heart failure, cardiac rehabilitation, and digital health technologies, using Boolean operators as follows:*(“heart failure” OR “cardiac failure” OR “HFrEF” OR “HFpEF” OR “diastolic heart failure”)* AND *(“cardiac rehabilitation” OR “heart rehabilitation” OR “cardiovascular rehabilitation”)* AND *(“artificial intelligence” OR “machine learning” OR “smartwatch” OR “wearable technologies” OR “wearable accelerometry” OR “ChatGPT” OR “digital health technologies” OR “telemedicine solutions” OR “telehealth” OR “Internet of Things” OR “algorithm” OR “gamification” OR “immersive virtual reality” OR “virtual coaching” OR “digital health platforms” OR “rehabilitation technologies” OR “virtual reality” OR “robotics” OR “exergaming”)*. The search covered publications from January 1, 2019, to December 31, 2024. The search was conducted on December 20, 2024. In total 172 documents met the selection criteria. The inclusion criteria were: participants diagnosed with heart failure, including HFrEF and HFpEF; eligible for cardiac rehabilitation and secondary prevention; study of types of technologies for rehabilitation of patients with heart failure (HF); randomized controlled trial, prospective intervention studies, systematic reviews, qualitative studies, cross-sectional studies, pilot studies, technology development, descriptive studies, longitudinal studies, observacional studies, predictive studies, experimental studies, comparative studies and case-control studies. The exclusion criteria were languages other than English; studies where results from patients with chronic diseases other than HF are not reported separately from the results pertaining to patients with HF and unspecified type of technology used in CR. The initial systematic literature search resulted in 172 articles on HF. The final selection of relevant articles comprised 59 articles. Figure 1 outlines the screening process. The following information was extracted from all included studies and is summarized in Figure 1: reference details, study design, sample size, technology employed, and reported outcomes. The literature search covered publications from January 1, 2019, to December 31, 2024, and was conducted on December 20, 2024. A total of 172 records were initially identified. Studies were considered eligible if they included participants diagnosed with heart failure, including HFrEF and HFpEF, and addressed the use of digital or technological solutions in cardiac rehabilitation and secondary prevention. A broad range of study designs was considered, including randomized controlled trials, prospective intervention studies, systematic and narrative reviews, qualitative and cross-sectional studies, pilot studies, technology development reports, descriptive and longitudinal studies, observational and predictive studies, as well as experimental, comparative, and case–control studies. Publications in languages other than English, studies that did not report heart failure–specific results separately, and studies in which the type of technology used in cardiac rehabilitation was not clearly specified were excluded. Following title and abstract screening and a focused full-text assessment, 59 articles were retained for inclusion in this review. The initial study selection was performed by the primary author, and the second author independently reviewed the selected articles to ensure consistency and relevance to the scope of the review. Figure 1 illustrates the study identification and selection process. From the included studies, key information was extracted to support a narrative synthesis, including reference details, study design, sample characteristics, type of technology employed, and reported outcomes.

## 5 Results

This review identified 59 studies investigating the use of digital technologies in cardiac rehabilitation (CR) for patients with heart failure. The included articles were grouped into nine categories according to the type and relevance of the technology addressed: platforms and websites (2, 7–11); mobile health (mHealth) applications (5, 12–19); artificial intelligence and machine learning approaches (20–24); wearable devices (3, 25–31); telehealth solutions (4, 32–52); assistive robots (53–55); smart hydration bottles (56); virtual reality and game-based interventions (57–59); and virtual assistants (60, 61). Each category reflects a distinct technological approach applied to CR, highlighting the diversity and innovation within the field. The distribution of technologies is summarized in Table 1. Telehealth was the most frequently studied technology (37.2%), followed by mHealth (15.3%), wearable devices (13.5%), platforms/websites (10.2%), artificial intelligence and machine learning (8.5%), assistive robots (5.1%), virtual reality and games (5.1%), virtual assistants (3.4%), and smart hydration bottles (1.7%). Regarding study design, randomized controlled trials (RCTs) constituted the largest proportion of the included studies, accounting for 23.7% of the total, followed by randomized controlled trial protocols (RCT Protocols) at 11.8%. Other study designs included cross-sectional studies (CSS) (10.2%), prospective intervention studies (PIS) (8.5%), pilot studies (PS) (8.5%), technology development studies (TD) (6.7%), systematic reviews (SR) (5.1%), qualitative studies (QS) (5.1%), experimental studies (ES) (5.1%), descriptive studies (DS) (5.1%), longitudinal studies (LS) (3.4%), observational studies (OS) (3.4%), comparative studies (CS) (1.7%), and case–control studies (CCS) (1.7%). Across all included studies, a total of 6,688 patients with heart failure (HF) were assessed, in addition to four cardiologists and five designers, primarily involved in studies focused on technology development and usability evaluation.

**Table 1:** Main results related to the technologies identified in cardiac rehabilitation (CR) of patients with heart failure (HF).

**Digital technologies**	**Reference/year/study design/sample size**	**Examples**
Platforms/websites	• (7) 2019/TD/n/a/; • (8) 2019/PIS/38; • (9) 2019/TD/59; • (2) 2020/RCT/n/a; • (10]2023/RCT Protocol/108; • (11) 2024/CSS/90.	Lalthanthuami (11) assessed the acceptance of a digital platform during the post-discharge period among 90 patients with HF. Results indicated a significant gap in adherence to physical exercise routines, although patient receptivity to mobile technologies for self-care support was high. These findings underscore the importance of developing user-centered digital interventions to enhance remote management of HF patients in resource-constrained settings.
mHealth	• (12) 2021/RCT Protocol/30; • (13) 2022/PIS/220; • (14) 2022/RCT/30; • (15) 2022/PS/164; • (16) 2022/SR/na; • (17) 2023/RCT/100; • (18) 2023/RCT/132; • (19] 2023/CSS/290; • (5) 2024/QS/25.	Saleh (18) evaluated the impact of a home-based mobile application intervention on physical activity levels and health-related Qol in 2 groups [control group [CG] and intervention group [IG]] of patients with HF over an 8-week period. Both groups received the same educational guidance; however, the IG additionally used a mobile application at home. The IG demonstrated greater improvements in physical activity levels and QoL compared to the CG. These findings suggest that home-based mHealth interventions can enhance physical activity and may play a significant role in promoting better outcomes for patients with HF.
AI/ML	• (20) 2020/TD/n/a • (21) 2021/ES/n/a; • (22) 2023/PS/50; • (23) 2023/RCTProtocol/64; • (24) 2024/QS/4	Filos (22) developed a machine learning-based methodology to predict the adherence of patients with HF to home-based cardiac rehabilitation (HBCR) programs with remote monitoring. A total of 50 HF patients were monitored using wearable devices during exercise sessions over a 6-month period. The primary innovation of this study lies in the application of ML techniques that combine patient characteristics, collected prior to the initiation of HBCR programs, with data gathered during a brief familiarization phase, enabling highly accurate long-term adherence predictions. The findings suggest that the proposed methodology can be generalized to other telerehabilitation programs, assisting healthcare professionals in enhancing personalized recruitment strategies and optimizing resource allocation.
Werables devices	• (25) 2019/TD/n/a; • (26) 2021/LS/41; • (3) 2021/SR/n/a; • (27) 2022/DS/n/a; • (28) 2023/LS/62; • (29) 2023/SR/n/a; • (30) 2024/OS/303; (31) 2024/RCT/80.	Lin (25) developed and evaluated an intelligent exercise guidance system based on smart clothing, integrating real-time cardiac monitoring, musical beat-guided feedback, and personalized running cadence adjustments to optimize exercise effectiveness. The smart clothing-based system demonstrated high efficacy in accurately collecting ECG signals, providing real-time heart rate (HR) monitoring, and delivering personalized guidance through musical beats. The integration of regression models to adjust running cadence enabled users to maintain their HR within recommended target zones, thereby optimizing exercise performance. This system shows significant potential for applications in CR programs and broader health promotion initiatives.
Telehealth	• (32) 2019/RCT/120; • (33) 2019/PS/4; • (34) 2019/PS/n/a; • (35) 2020/RCT/288; • (36) 2020/CSS/34; • (37) 2022/CSS/81; • (38) 2021/CSS/18; • (39) 2021/RCT/782; • (40) 2021/RCT/386; • (41) 2021/OS/386; • (42) 2021/DS/56; • (43) 2023/RCT/1538 • (44) 2023/RCT/61; • (45) 2023/RCT/50; • (4) 2023/ES/64; • (46) 2023/CSS/117; • (47) 2023/PIS/49; • (48) 2024/DS/49; • (49) 2024/CS/180; • (50) 2024/RCT/75; • (51) 2024/RCT/122; • (52) 2024/RCT Protocol/70.	Tsai (37) evaluated the effectiveness of a 6 month home-based cardiac telerehabilitation (HCTR) program in improving functional capacity and reducing hospital readmission rates in elderly patients with HF. A total of 81 HF patients were divided into two groups: an intervention group (IG) and a control group (CG). The IG participated in a supervised HCTR program that included telemonitored physical exercise sessions and daily entries into a mobile cardiac health management application, with data transmitted to a hospital database. The CG received only usual care. The HCTR program proved to be safe and effective in improving functional capacity and cardiac function, as well as in reducing hospital readmission rates among elderly HF patients. These findings suggest that telerehabilitation interventions may represent a viable alternative to traditional CR programs, particularly for the elderly population.
Assistive robots	• (53) 2022/PIS/52; • (54) 2023/PIS/n/a; • (55) 2024/RCT/90.	Hirashiki (55) evaluated the clinical safety and effectiveness of a balance exercise assist robot (*BEAR*), which incorporates video game elements to improve posture and balance, in elderly patients with HF. In this study, 90 patients were randomized into two groups over a 4-month period: the control group (CG) underwent conventional resistance training, while the intervention group (IG) performed training using the *BEAR* system. The IG demonstrated superior improvements in motor function and nutritional risk scores compared to the CG. CR using the *BEAR* robot was found to be safe and comparable to conventional resistance training
Smart hydration bottle	(56) 2024/RCT Protocol/n/a.	Park (56) presented RCT protocol utilizing a multi-technology approach, combining wearable devices (Fitbit), the *(activPAL*) mobile application, a smart hydration bottle (*HidrateSpark)*, and personalized text messaging. This protocol represents a promising and cost-effective strategy to reduce sedentary time, with the potential to improve cardiometabolic health and decrease the risk of recurrent cardiovascular events.
VR/Games	• (57) 2020/ES/65; • (58) 2023/RCT Protocol/66; • (59) 2024, PS/29.	Suzuki (59) valuated the effectiveness of a gamification program based on behavioral economic theories in 29 patients with HF. The objective was to increase daily step counts using a smartphone equipped with a gamification application and an automatic step data collection system integrated with *Google Fit*, over a total period of 7 weeks. The results demonstrated a significant increase in step counts, suggesting that this approach could serve as a useful tool to promote physical activity and support secondary prevention in cardiovascular disease.
Virtual assistants	• (60) 2022/QS/11; • (61) 2023/CCS/30.	Lăcraru (61) evaluated the efficacy of the virtual assistant *vCare* in the remote rehabilitation of 30 patients with HF, who were divided into 3 groups: G1 underwent home-based rehabilitation assisted by vCare; G2 participated in conventional in-clinic rehabilitation; and G3 received only standard discharge instructions, over a 3-month period. Clinical outcomes showed significant improvements in both G1 and G2 compared to G3. The *vCare* system demonstrated benefits comparable to conventional programs and appears to be a promising solution to enhance continuity of care and treatment adherence in HF patients, particularly in settings with limited access to in-person services.

Table 1 presents the main results related to the identified technologies in the CR of patients with HF.

## 6 Discussions

The findings of this review highlight the diversity of digital technologies at different stages of development and validation for the management of patients with heart failure (HF). An increasing number of studies have explored remote monitoring approaches, particularly during and following the COVID-19 pandemic, suggesting their role as a complementary alternative to traditional outpatient cardiac rehabilitation (CR) programs. Among the technologies identified, digital platforms have been reported to support patient engagement and facilitate clinical follow-up (7), while step-count monitoring has been described as a potential indicator of functional capacity in this population (2). Platforms such as vCRP (8) have been designed to replicate core elements of face-to-face care—such as education, support, and motivation—indicating that virtual care models may be feasible for CR delivery, especially in geographically underserved settings. In this context, the Internet of Things (IoT) has emerged as a key enabling framework, allowing connectivity between wearable devices, smart sensors, and digital platforms to support continuous and remote physiological data collection. Participatory design approaches, such as those used in the development of the HeartPortal platform, have been associated with favorable usability evaluations and alignment with user needs (9). Similarly, studies by Chimura and Lalthanthuami reported acceptable safety profiles and feasibility of supervised telerehabilitation platforms among older adults (10, 11). Mobile health (mHealth) technologies have also been described as feasible components of CR programs, particularly when individual preferences, educational support, and trust in digital systems are considered (5, 12, 14, 18). These tools may facilitate the collection of objective data that can support the personalization of exercise programs (13, 14). Digital biomarkers have been proposed as emerging tools for more detailed monitoring of clinical events (19). Some studies have reported associations between digital interventions and outcomes such as treatment adherence, program completion, and hospital readmissions (15); however, these findings should be interpreted cautiously given the heterogeneity of study designs and outcome measures. Notably, a critical review of mobile applications identified that only a small proportion adhered to evidence-based clinical guidelines, emphasizing the need for improved regulatory standards and clinical validation (16). Digital CR interventions have generally been associated with high levels of patient acceptance, influenced by factors such as perceived usefulness, ease of use, and digital trust (19). Studies involving artificial intelligence (AI) and machine learning (ML) technologies suggest their potential to support clinical workflows, for example by assisting in the prioritization of patient messages without compromising safety (20). While conversational AI tools such as ChatGPT may provide general information on HF, their application in clinical decision-making should remain under professional supervision (24). ML-based models have also been explored for risk prediction and early identification of heart disease, although further validation is required before widespread clinical adoption (21, 23). Wearable technologies—including smartwatches, step counters, and smart garments—have been widely investigated as part of the IoT ecosystem, enabling non-invasive and continuous data collection in home settings (3). Studies have explored the use of smart clothing (25), wearable cameras (27), and behavioral digital biomarkers such as GENEActiv (30). While these technologies show promise for real-time monitoring of patient-reported and functional outcomes, economic constraints and accessibility issues remain important barriers to large-scale implementation, underscoring the need for further randomized clinical trials and cost-effectiveness analyses (31). Telehealth emerged as the most extensively studied modality, appearing across a wide range of randomized and non-randomized studies (32, 35, 39, 40, 43–45, 49–51, 61). Several studies suggest that telehealth-based CR programs can achieve outcomes comparable to conventional face-to-face models in terms of adherence, motivation, and physical activity levels (33, 48), while also supporting disease awareness and digital health literacy (34, 52). Hybrid CR models have been described as feasible, and multiple studies have reported improvements in quality-of-life measures following telerehabilitation interventions (38, 41, 42, 47), although variability across study designs warrants cautious interpretation. Usability challenges persist across digital CR solutions, reinforcing the importance of user-centered design approaches (36, 43). Assistive robotic technologies have been explored as supportive tools for balance training in older HF patients; however, high costs and cognitive demands currently limit their scalability (53, 55). These systems may facilitate patient–provider interaction and support autonomy, though broader implementation remains constrained (54). Virtual reality (VR) has been investigated as a supportive psychological and motivational tool in CR settings (58), with some studies reporting reductions in perceived dyspnea and improved engagement during hospitalization (57). Virtual assistant technologies have also been explored as alternative support mechanisms for patients with limited access to traditional CR programs, suggesting potential benefits in functional and behavioral outcomes that require further confirmation (60, 61). Overall, the integration of emerging digital technologies—particularly within IoT-enabled ecosystems—represents a promising direction for the evolution of cardiac rehabilitation toward more personalized, continuous, and accessible models of care. Nevertheless, the current evidence base remains heterogeneous and largely exploratory, highlighting the need for well-designed clinical trials, standardized outcome measures, and robust validation frameworks before these technologies can be fully integrated into routine HF management.

## 7 Conclusions

This review highlights the broad spectrum of emerging digital technologies applied to the management of heart failure, with particular emphasis on the increasing relevance of Internet of Things (IoT)–based systems. Remote monitoring solutions—especially those expanded during the COVID-19 pandemic—have been increasingly explored as complementary approaches to conventional cardiac rehabilitation, enabling continuous physiological data collection and remote clinical follow-up. Digital platforms have been reported to support patient engagement and facilitate care delivery, while mobile and wearable devices integrated within IoT ecosystems may assist in monitoring physical activity and adherence outside clinical settings. Artificial intelligence and machine learning approaches, when combined with IoT infrastructures, have been investigated as scalable tools to support clinical workflows and risk stratification, although their real-world clinical impact remains under evaluation. Similarly, technologies such as virtual reality, assistive robotics, and virtual assistants have emerged as potential adjuncts to traditional rehabilitation models, particularly in addressing motivational and psychological aspects of care. Despite these technological advances, significant challenges persist, including the limited availability of large-scale validation studies, economic and accessibility barriers, usability concerns—especially among older adults—and variable adherence of digital applications to evidence-based clinical guidelines. Collectively, the findings of this review suggest a growing opportunity for the development of IoT-enabled digital health solutions that are accessible, interoperable, and grounded in robust clinical evidence. Future research should prioritize rigorous clinical validation, long-term outcome assessment, data security, user-centered design, and sustainable integration of these technologies into routine heart failure care.

## 8 Limitations

This review has some limitations, including the heterogeneous nature of the included studies, the absence of a formal risk-of-bias assessment, and the exploratory character of much of the available evidence, which should be considered when interpreting the findings.
